# 新型量子点基分子印迹荧光传感器在快速检测中的应用

**DOI:** 10.3724/SP.J.1123.2021.02025

**Published:** 2021-08-08

**Authors:** Jiaxin MA, Ziru LIAN, Cheng HE, Jiangtao WANG, Rencheng YU

**Affiliations:** 1.山东大学海洋学院, 山东 威海 264209; 1. Marine College, Shandong University, Weihai 264209, China; 2.中国海洋大学化学化工学院, 山东 青岛 266100; 2. College of Chemistry and Chemical Engineering, Ocean University of China, Qingdao 266100, China; 3.中国科学院海洋研究所海洋生态与环境科学重点实验室, 山东 青岛 266071; 3. CAS Key Laboratory of Marine Ecology and Environmental Sciences, Institute of Oceanology, Chinese Academy of Sciences, Qingdao 266071, China

**Keywords:** 量子点, 分子印迹技术, 荧光传感器, 快速检测, 痕量分析, quantum dots (QDs), molecular imprinting technology (MIT), fluorescence sensor, rapid detection, trace analysis

## Abstract

作为一种新型荧光纳米材料,量子点具有十分优异的光学特性,是分析化学、生物科学、医学等领域研究的热点标记材料。分子印迹聚合物是能够进行特异性识别和选择性吸附的“仿生”材料,它易于制备且具有较好的重现性和稳定性,因而分子印迹技术已成为具有广阔应用前景的识别技术。量子点基分子印迹荧光传感器结合了量子点和分子印迹技术的优势,由于其高选择性和高灵敏度,在环境监测、食品检测、生物分析等领域得到快速发展。但该传感器在应用中也还存在亲水性不足、识别单一、便携性不足等问题。该文引用了近5年来发表在American Chemical Society、Elsevier等数据库约20篇相关文献,对量子点基分子印迹荧光传感器的构建及该传感器在快速检测分析痕量物质中的应用进展进行了综述。首先根据荧光光谱图中发射峰个数的不同分别介绍了3种量子点基分子印迹荧光传感器的类型及相关识别机理,其次根据待测物的不同归纳介绍了近五年来该传感器在离子、有机小分子、生物大分子等检测分析中的最新研究进展,最后对当前该传感器在制备及应用中仍存在的问题进行了总结并对其发展趋势进行了展望。

## Abstract (en)

A critical need in analytical chemistry is the efficient fabrication of selective and sensitive sensors to detect trace analytes in complicated samples. In recent years, fluorescence analysis has been widely used in environmental research and the life sciences due to its high sensitivity and simple operation. Quantum dots (QDs) are a new type of fluorescent nanomaterials. Owing to the quantum confinement effect, QDs possess excellent optical properties such as strong anti-bleaching ability, a narrow excitation and emission band, and tunable emission wavelength. As a hot labeling material, QDs are suitable for use in surface-modified analytical sensors employed in fields such as analytical chemistry, biology, and medicine. However, QD materials have a notable disadvantage, in that the actual sample matrix may contain some interferents with luminescent responses similar to those of the target; this decreases the selective ability of the fluorescence sensor. The surface modification of QDs via the molecular imprinting technique (MIT) is a promising solution to overcome this drawback. Molecularly imprinted polymers (MIPs) are a kind of “bionic” material that can carry out specific recognition and selective adsorption and hence, possess the unique properties of recognition specificity, structural predictability, good reproducibility, and excellent stability. Accordingly, MIPs have been widely employed in sensors as well as for drug delivery, catalysis, and solid phase extraction. Notably, QD-based molecularly imprinted fluorescence sensors combine the advantages of QDs and the MIT. Owing to their specific selectivity and high sensitivity, such sensors have been extensively developed for environmental monitoring, food detection, and biological analysis. However, there remain challenges associated with the preparation and application of the sensors: (i) single recognition: it is important to develop a composite sensor that can detect multiple target analytes from the actual samples at the same time during practical application; (ii) poor hydrophilicity: the actual sample is usually a liquid matrix; hence, it is imperative to determine an approach for improving the hydrophilicity of the sensor; (iii) the accuracy of fluorescence response and the resolution of visual detection need to be further improved; (iv) imprinting: it remains challenging to imprint biological macromolecules, viruses, and bacteria. Thus far, many researchers have made progress with regard to the preparation and application of the sensors. Accordingly, this work reviews approximately 20 papers published by the American Chemical Society, Elsevier, and other databases in the last five years to highlight progress in novel preparation methods and practical applications of QD-based molecularly imprinted fluorescence sensors for the sensitive analysis and rapid detection of trace substances. First, according to the different numbers of emission peaks in the fluorescence spectrum, three kinds of QD-based molecularly imprinted fluorescence sensors are introduced and the related recognition mechanisms are explained. Second, according to the different substances to be detected, this mini-review summarizes the latest research progress in sensors for the detection of ions, organic small molecules, biological macromolecules, as well as for the analysis of bacteria and viruses. Finally, existing challenges associated with the preparation and application of the sensors, as well as future development trends, are discussed.

量子点(quantum dots, QDs)是一种新型半导体荧光纳米材料,其结构决定了它具有优异的光化学稳定性、激发谱宽而发射谱窄以及较长的荧光寿命等特点^[1]^。目前研究较多的量子点主要包括镉系量子点如CdSe、CdS、CdTe,铟系量子点如InGaAs、InP、InAs,硅量子点,碳量子点等,其中碳量子点(carbon dots, CDs)因具有高生物相容性和低细胞毒性而备受关注。量子点材料异于其他荧光材料的优异光学特性使其极适合作为荧光探针用于传感分析,量子点荧光传感器灵敏度高、易于操作,近年来在环境监测、细胞成像、疾病诊断和治疗等领域发展迅速。然而其存在一个突出的缺陷,即实际样品基质可能存在一些与目标待测物发光响应性质相似的物质,导致荧光传感器的选择能力降低^[2]^。为解决这一问题,研究人员将分子印迹技术(molecular imprinting technique, MIT)引入荧光传感器。分子印迹技术是通过模拟天然分子识别现象,人工合成对模板分子能够进行特异性和选择吸附的高分子功能材料即分子印迹聚合物(molecularly imprinted polymers, MIPs)的一种新技术。在早期研究应用中,MIPs通常用于萃取/分离,检测分析仍需联用色谱/质谱等手段,无法实现现场的快速检测^[3]^。分子印迹荧光传感器以MIPs为识别元件、QDs为响应元件,结合了QDs和MIT的优势,具有高选择性和高灵敏度,是一种易于操作的快速检测新手段。本文就此介绍了目前基于量子点荧光材料的分子印迹传感器的3种类型,并综述了近5年来该检测技术的最新研究应用结果。

## 1 量子点基分子印迹荧光传感器的类型

分子印迹荧光传感器用于分析检测各种物质的过程大体为:当待测物质被分子印迹聚合物立体孔穴特异性吸附时,荧光材料会发生光诱导电子转移(photoinduced electron transfer, PET)、荧光共振能量转移(fluorescence resonance energy transfer, FRET)等物理化学变化,从而导致其荧光信号发生相应变化,利用荧光分光光度计检测得到的荧光光谱图中靶敏信号的发射峰会发生峰值的改变,通过制得标准曲线即可检测待测物质。根据荧光光谱图中发射峰个数的不同,分子印迹荧光传感器大体可以分为3种:单发射分子印迹荧光传感器、双发射分子印迹荧光传感器和三发射分子印迹荧光传感器。

### 1.1 单发射分子印迹荧光传感器

单发射分子印迹荧光传感器指的是合成及检测过程仅存在一种荧光物质,在特定激发波长下荧光光谱图中仅有一个发射峰为靶敏信号,以其在吸附过程中的变化值作为衡量样品中待测物质含量的标准。传统意义上的单发射分子印迹荧光传感器通常由一锅法制备,印迹位点多包埋于内部,结合模板分子速率慢且洗脱困难,近年来基于表面印迹的核-壳型MIPs传感器发展迅速。Amjadi等^[4]^以硅基为载体,通过反相微乳液法嵌入CdTe QDs,合成了一种基于PET原理检测氯霉素(chloramphenicol, CAP)的新型荧光纳米传感器。由于CAP的紫外-可见吸收带接近CdTe QDs的带隙,因此在识别过程中,QDs可作为电子供体将电子转移到CAP分子的最低未占有分子轨道(lowest unoccupied molecular orbital, LUMO),导致CdTe QDs在550 nm处的荧光猝灭。

### 1.2 双发射分子印迹荧光传感器

双发射分子印迹荧光传感器指的是合成及检测过程存在两种荧光物质,在特定激发波长下荧光光谱图中有两个发射峰(一个为参比信号,一个为靶敏信号),在吸附过程中靶敏信号的峰值发生变化而参比信号的峰值保持不变或发生与前者相反的变化,以吸附过程中二者比值作为衡量样品中待测物质含量的标准,因此较上述单发射传感器而言,双发射传感器具有自我校正功能,可以减少或消除人为及机器带来的误差。双发射分子印迹荧光传感器目前主要包括两类,一类是待测物本身带有荧光,如Xu等^[5]^基于FRET识别原理构建了一种用于检测阿霉素的分子印迹荧光传感器。由于其发射光谱与阿霉素的吸收光谱重叠,当两者在空间上距离<10 nm并接收一定波长的光照射后,CDs荧光供体与阿霉素受体之间发生荧光共振能量转移,荧光供体的激发能转移到荧光受体上继而使其被激发,导致CDs荧光逐渐猝灭而阿霉素荧光逐渐增强,实现了对阿霉素快速地选择性分析。另一类是待测物不带有荧光,如苏立强团队^[6]^以CDs和CdSe QDs组成的双荧光体系结合分子印迹技术制备比率型传感器,在吸附过程中参比CdSe QDs由于包埋在SiO_2_中荧光强度几乎不变,外层CDs荧光强度随着待测物浓度而变化。

### 1.3 三发射分子印迹荧光传感器

三发射分子印迹荧光传感器指的是合成及检测过程存在3种荧光物质,在比率型荧光传感器基础上增加一个新的荧光信号,通过三元发射扩大可视化窗口、提高裸眼检测精确度,目前有关研究相对较少。Yang等^[7]^分别制备以绿色和红色量子点(g-QDs和r-QDs)为荧光材料、叶酸为模板分子的核-壳型分子印迹荧光传感器,然后以适当的比例混合得到基于PET的三重发射分子印迹荧光传感器。在吸附过程中,叶酸的羟基和氨基与3-氨丙基三乙氧基硅烷的氨基之间形成氢键,叶酸具有接近g-QDs@MIPs和r-QDs@MIPs带隙的紫外吸收峰,因此在QDs导带处的激发电荷易转移到叶酸的LUMO而无法回到QDs价带,进而引起QDs的绿色荧光和红色荧光逐渐猝灭而叶酸荧光增强。通过条件优化,使g-QDs和r-QDs之间的猝灭速率差异被扩大,以获得更宽范围和丰富的荧光颜色演化,这种出色的可视化能力使得仅通过便携式紫外灯裸眼准确读出叶酸浓度成为可能。

## 2 量子点基分子印迹荧光传感器在快速检测中的应用

量子点基分子印迹荧光传感器已经被广泛应用于环境、食品、生物等领域进行痕量物质甚至超痕量物质的检测分析。根据被检测物质性质,我们将从离子检测、有机小分子检测和生物大分子检测3个方面来阐述其在多个领域的实际应用。

### 2.1 离子的分析检测

离子印迹是印迹聚合物的一个重要分支,由于环境中残留重金属对于人类生命安全危害极大,因而目前对于离子印迹的研究多以铅(Pb)、汞(Hg)、铜(Cu)等重金属离子为模板,通过其特有的金属配位作用制备得到具有特异性识别能力的离子印迹聚合物(ion imprinted polymers, IIPs),与灵敏荧光材料结合即可实现对目标离子的选择性富集、快速痕量检测。Patra等^[8]^构建了一种灵敏检测Ag^+^的新型纸基离子印迹荧光传感器,将荧光素染料用作制备CDs的前驱体,该传感器具有低毒性、高稳定性和良好的量子产率。该CDs@MIPs改性滤纸条已成功地用于活细胞成像和Ag^+^的细胞内分析,响应时间<10 s。针对多种离子同时检出问题,Xu课题组^[9]^首次提出了同时检测两种金属离子的双参比离子印迹荧光传感器,以分别对Fe^3+^和Cu^2+^有响应的氨基修饰CDs和羧基修饰CdTe QDs为荧光团,通过一锅法合成制备了介孔结构双模板双参比离子印迹荧光探针。当检测Fe^3+^时,以CDs为参比,CdTe QDs猝灭;而当检测Cu^2+^时,以CdTe QDs为参比,CDs猝灭。使用这种双模板双参比策略,可以在真实水样中同时检测Fe^3+^和Cu^2+^。在保持IIPs灵敏度和选择性的同时,制备的双参比荧光传感器可以同时检测两种离子,大大提高了检测效率,同时该工作创新了印迹传感器领域的信号输出模式,为高通量检测带来了技术进步。此外,同种离子具有不同价态且常同时存在,针对这一问题,Jinadasa等^[10]^成功研制了一种室温荧光化学传感器并应用于鱼类样品中无机砷(As^3+^和As^6+^)的选择性检测和定量分析。该方法操作简单,仅需较短的分析时间,可获得良好的灵敏度和精密度。

### 2.2 有机小分子的分析检测

在食品残留与环境污染问题中主要目标检测物通常为有机小分子,因而目前分子印迹荧光传感器研究较多的方面是针对有机小分子的痕量分析,但在传感器制备技术上仍然存在一定问题,近年来研究人员也提出了一些新策略。当前,制备分子印迹荧光传感器时主要还是采用包埋法将荧光材料裹于硅基中,该方法会大大降低荧光材料的荧光强度从而影响传感器灵敏度。针对此问题,Yu等^[11]^以甲基丙烯酸2-氨基乙酯盐酸盐作为表面活性剂修饰羧基CdTe QDs,并以被修饰QDs为支撑和荧光信号源,通过自由基聚合一锅法直接合成了超薄4-硝基苯酚印迹层;该方法避免了合成及使用基质材料包埋的过程,从而实现对于海水和湖水样品中4-硝基苯酚的高检测灵敏度。由于传统硅基材料内聚扩展阻力会影响吸附位点对于待测物的识别,Amjadi等^[12]^设计了一种双发射介孔结构分子印迹荧光传感器,用于杀菌剂烯唑醇的特异性识别和检测。CdTe/CdS QDs被封装在SiO_2_介孔中,识别过程中CdTe/CdS QDs的荧光被选择性猝灭,介孔的存在降低了传质阻力、提高了位点选择性,对烯唑醇的识别特异性明显优于其类似物。由于实际应用时的需求,多种分析物的同时检测引起了研究人员极大的兴趣,Wei等^[13]^通过在两种不同颜色的QDs表面分别以不同模板分子制备MIPs,而后将所得MIPs等比例混合,建立了一种同时检测去甲肾上腺素和肾上腺素的方法,这项工作为QDs@MIPs实际应用中同时检测不同分析物提供了新的思路。为了提高监测设备的便携性,Sari等^[14]^利用纸基纳米复合体系制备了一种新型光传感材料用于三丁基锡化合物的检测。他们以硝酸纤维膜为支撑,采用微乳液聚合技术合成印迹纳米颗粒,然后将石墨烯QDs耦合固定在纳米粒子表面。结果表明,其对海水中的三丁基锡化合物具有良好的选择性、高灵敏度和低检出限。由于检测基质通常为液体环境,发展亲水性传感器势在必行。Xu等^[15]^在制备过程中引入了亲水聚甘油单甲基丙烯酸酯刷,通过自由基聚合构建了“亲水开启型”比率传感器,可直接用于未被稀释的纯羊奶和牛奶中除草剂2,4-二氯苯氧乙酸的快速分析测定。传统使用的量子点如CdTe QDs等含有重金属元素,对于环境并不友好,新型绿色CDs应运而生,如Liu等^[16]^以桂花叶为碳源,分别采用乙醇和聚乙烯亚胺溶液萃取制备了两种不同发射峰的高荧光CDs,并分别作为参比和靶敏信号,构建了比率荧光传感器用于四环素的高选择性、高灵敏度检测。此外,Zhang等^[17]^成功制备了基于量子点接枝有机框架的双功能分子印迹光聚合物(molecularly imprinted optopolymer, MIOP),使得通过光传感和固相萃取-高效液相色谱同时检测酪胺成为可能。在优化条件下,酪胺含量在35~35000 μg/kg时,光传感方法的相对荧光强度线性增加,检出限为7.0 μg/kg;而对于固相萃取-高效液相色谱,线性范围为20~2000 μg/kg,检出限为5.0 μg/kg。

### 2.3 生物大分子的分析检测

生物大分子、细菌和病毒等由于自身尺寸大、易变性且纯天然模板难获取,一直以来都是分子印迹技术的难点,尽管困难重重,但是应用前景广阔。新型印迹方法如表面印迹、金属螯合印迹、抗原印迹等的提出使生物大分子的分析检测得到了一定发展,这其中对于蛋白质的研究较多。陈令新课题组^[18]^构建了基于QDs的选择性灵敏检测藻蓝蛋白的印迹荧光纳米传感器,他们将以巯基乙酸和谷胱甘肽共同作为稳定剂修饰的QDs作为功能单体,通过多巴胺自聚合有效地简化了印迹过程,同时识别位点更加容易接近,该纳米传感器在结合藻蓝蛋白后16 s内就显示出明显的荧光衰减,提供了一种通用的蛋白质印迹策略。该课题组^[19]^还制备了一种热敏比率印迹荧光纳米传感器,表现出良好的温度响应、高灵敏度和选择性,实现了基于FRET的温度调节传感识别检测藻蓝蛋白。此外,为了扩大可视化窗口,使裸眼检测蛋白成为可能,Yang等^[20]^通过印迹后混合适当比例的蓝/绿/红发射牛血红蛋白(bovine hemoglobin, BHb)印迹材料,构建了一种新型的三重发射荧光传感器,识别时伴随着宽范围荧光颜色演变,覆盖了绿色-红色-蓝色窗口,实现了对BHb的多重可视化检测。以上对蛋白质的检测均在体外进行,Cecchini等^[21]^报道了一种新型体内识别蛋白质的分子印迹荧光传感器,以人血管内皮生长因子的83~91位氨基酸为模板、CdTe QDs为荧光信号源构建印迹荧光纳米传感器,可在体内肿瘤异种移植实验中定位过度表达人类表皮生长因子的肿瘤块,该研究为印迹荧光传感器体内靶定分泌因子提供了新思路。除蛋白质外,在DNA方面的印迹也取得了一定进展。Arslan等^[22]^报道了一种适用于dsDNA快速检测的比率印迹荧光传感器,分别以Mn-ZnS QDs和阳离子染料孔雀石绿为荧光信号源,识别dsDNA时孔雀石绿分子从QDs@MIPs表面逃逸并嵌入dsDNA、荧光增强,同时QDs@MIPs的实时荧光开启,实现了dsDNA的快速选择性检测,检出限为19.48 ng/mL。在细菌检测方面,Zhao等^[23]^建立了一种基于分子印迹荧光传感器对单增李斯特菌的有效识别方法。该传感器以经过修饰的壳聚糖(NAC@CdTe QDs)为功能单体,制备过程中通过Pickering乳液聚合与分子印迹技术相结合,制备的印迹荧光传感器能够通过三维孔穴选择性捕获目标细菌,并成功应用于牛奶和猪肉样品中单增李斯特菌的分析。在病毒检测方面,Luo等^[24]^报道了一种混合分子印迹荧光传感器,用于同时检测甲型肝炎病毒(hepatitis A virus, HAV)和乙型肝炎病毒(hepatitis B virus, HBV)。该传感器采用印迹后混合策略,分别以红色、绿色QDs为荧光材料,HAV、HBV为模板分子制备MIPs而后以适当比例混合。结果表明,对两种病毒的检测均达到了令人满意的选择性和灵敏度,HAV和HBV检出限分别为3.4和5.3 pmol/L。

## 3 总结与展望

目前量子点基分子印迹荧光传感器在环境监测、食品安全和生物分析等领域展现出巨大的优势和广阔的应用前景,但该传感器在制备、应用等方面仍存在着一定的问题:印迹过程需要用到大量模板分子,有些化合物价格昂贵,还有些化合物如代谢产物、生物活性物质等不易得到,这大大限制了分子印迹荧光传感器的制备和应用;如何实现对于生物大分子以及病毒、细菌的印迹仍是重大挑战;在实际应用检测时,对于一个样本需要同时检测两种或多种残留物质的情况十分普遍,开发出能够对样本中多种目标物质同时检测的复合型传感器,是该技术有待深入研究的一个重要方向。总之,对于量子点基分子印迹荧光传感器的相关研究开展时间并不长,这项技术还需要学者不断地探索和完善,以期获得更大的发展和应用。
